# Fast-track flipping: flipped classroom framework development with open-source H5P interactive tools

**DOI:** 10.1186/s12909-021-02784-8

**Published:** 2021-06-22

**Authors:** Judith Wehling, Stefan Volkenstein, Stefan Dazert, Christian Wrobel, Konstantin van Ackeren, Katharina Johannsen, Tobias Dombrowski

**Affiliations:** 1 Department of Otolaryngology, Head and Neck Surgery, Ruhr University Bochum, St. Elisabeth Hospital, 15, 44787 Bochum, Germany; 2 Department of Otolaryngology, Head and Neck Surgery, University Medical Centre, Göttingen, Germany

**Keywords:** Flipped classroom, Medical education, Otorhinolaryngology, Undergraduate education, Blended learning

## Abstract

**Background:**

The availability and popularity of laptops, tablet PCs and smartphones in private and work environments offers considerable potential for reasonably integrating blended learning formats into structured medical learning environments. The promising educational principle of the flipped classroom (FC) provides the opportunity to effectively combine e-learning and face-to-face teaching within a single framework. However, similar to most blended learning formats, the FC requires a solid groundwork of structured digitized learning content. As rearranging a whole curriculum is intense and time consuming, physicians occupied simultaneously in clinical practice and teaching may be confronted with a lack of time during this process.

**Methods:**

We developed two straightforward approaches to transforming a pre-existing, lecture-based otolaryngology curriculum into interactive videos within a Moodle learning management system. Special attention was given to reducing individual working time for medical professionals. Thus, while one approach was mainly guided by a medical professional to control the content-related quality of video processing, we investigated an alternative approach outsourcing work to a technician. Afterwards, the working time was analysed and compared. The resulting videos were revised with the H5P plugin for moodle to adjust the content where necessary.

**Results:**

We identified a fast-track approach for creating structured e-learning content suitable for flipped-classroom-based lectures, other blended learning formats, or even providing a whole curriculum online. The alternative approach significantly reduced working time for medical professionals but did not impair the content-related quality significantly.

**Conclusions:**

The use of H5P interactive tools via Moodle LMS provides a major procedural benefit by allowing the easy adjustment of pre-existing video material into suitable online content. Reasonably outsourcing work to technicians can significantly reduce the working time of medical professionals without decreasing the quality of learning content. The presented workflow can be used as a flexible approach for flipped classroom frameworks or other blended learning strategies where interactive videos are applicable.

## Background

Undergraduate clinical teaching is at least partially dependent on face-to-face-learning environments as medical education includes both theoretical and practical learning objectives. Even though e-learning focuses on the individual student and provides high accessibility due to the use of laptops, tablet PCs and smartphones, e-learning resources are poorly integrated into medical framework curricula [1]. Therefore, there is still much potential for reasonably combining e-learning and face-to-face teaching in structured medical blended learning environments [2]. The popularity of electronic learning approaches is at least partially driven by the option to use diversified teaching formats to develop innovative individual courses. For example, the student-centred educational principle of the flipped classroom (FC) is a promising model for medical education and has already been established for a broad range of medical education subjects [3–8]. In this context, students autonomously prepare by learning basic knowledge on the basis of videos, quizzes, etc., at home, and they subsequently meet with the lecturer to gain deeper insights and participate in problem-solving exercises [1, 4, 6, 9, 10]. Thus, the FC does not focus on passive knowledge transfer but on an active, problem-oriented interaction with consequences for clinical practice. Related literature is heterogeneous showing varying effects and effect sizes for the learning outcome and may often biased by increased teaching and learning activities in the experimental setting [9, 11, 12]. A recent meta-analysis showed a risk of bias in 36 of 37 included articles [13].

Previously published works agree that the development of a successful FC calls for a change in the whole framework of the curriculum since lecture content has to be filtered in terms of its suitability for flipped classrooms, rearranged and enriched with practical, problem-oriented aspects [1, 10, 14]. To date, most of the literature naturally focuses on learning outcome, curriculum structure and didactics [9]. Moreover, the preparation of (multimedia-based) theoretic lessons is intense and time-consuming as the teacher has to harmonise the self-directed learning phase with the in-classroom teaching. This increased working time for the teachers has already been reported and was e.g. documented by McLaughlin and colleagues as an increase of 127% [14]. Anyway, structured multimedia-based content represents the inevitable groundwork for the self-directed learning phase and thereby the FC framework [15]. Thus, it may be convenient to refer to pre-existing open educational resources for the self-directed learning phase. While this strategy could be useful for some selected blended learning environments, theoretic lessons created by a third party may work against a specific preparation for the face-to-face lessons of a FC and prevents from pointing out local focus areas [16]. Recent work of our group also showed that students may prefer educational material generated by the teacher [5].

In summary, there are several ideas why self-made multimedia content could be beneficial for the teacher in a blended learning approach, especially using the flipped classroom principle. If the ground level state is a classical, lecture-based curriculum, like it was in our case, this demand may force the teacher to start from scratch requiring technical expertise, resources and time as well as possibly impairing cost-effectiveness [17].

To address this problem, which is beyond the scope of most publications, we present a novel strategy for a fast-track transformation of a lecture-based curriculum into a flexible, flipped classroom-supported framework within a Moodle learning management system (LMS) accompanied by a detailed documentation and analysis of working time and content. Our approach is substantially supported by the HTML5 Plugin H5P to equilibrate the conducted dissection of the lectures into interactive video chunks that, to some extent, initially led to a loss of context. We especially focused on developing and investigating strategies for minimizing working time for medical professionals during the whole procedure. Therefore, an alternative approach, partially outsourcing work to technicians, was explored and compared to an approach processed by medical professionals with regards to content. The idea of outsourcing work to a technician was to accelerate the removal of inapplicable content by reducing the physician’s task to a binary “keep or discard”-decision In spite of focusing on an otolaryngology curriculum in this publication, the principle could likewise be transferred to other subjects.

## Methods

**Fig. 1:**
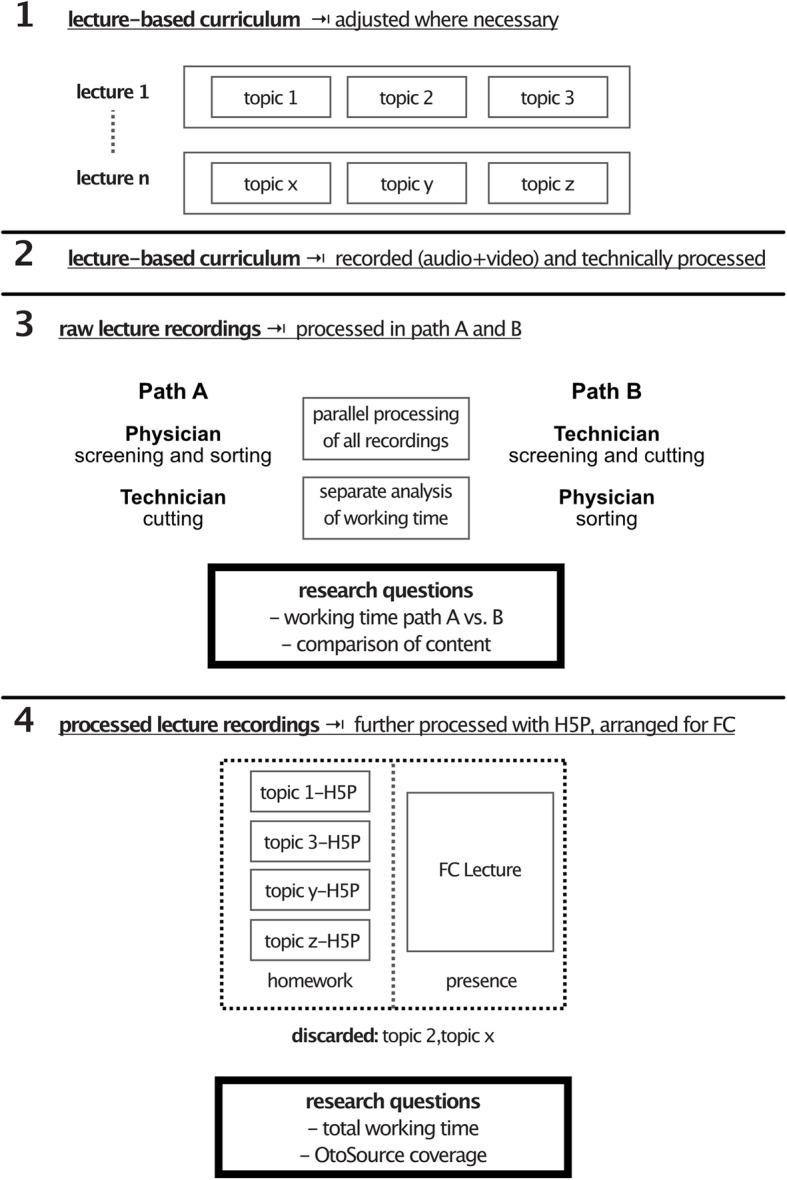
Workflow for lecture processing towards a flipped classroom framework. After filming the lecture, videos were screened by physicians (Path A) or technicians (Path B) and edited by technicians. Afterwards, the video material was sorted by a physician and enriched with H5P tools. The resulting material was used for a flipped classroom curriculum

For the transformation of our complete lecture-based curriculum into online learning material we followed a four-step procedure (Fig. 1).

**Fig. 2:**
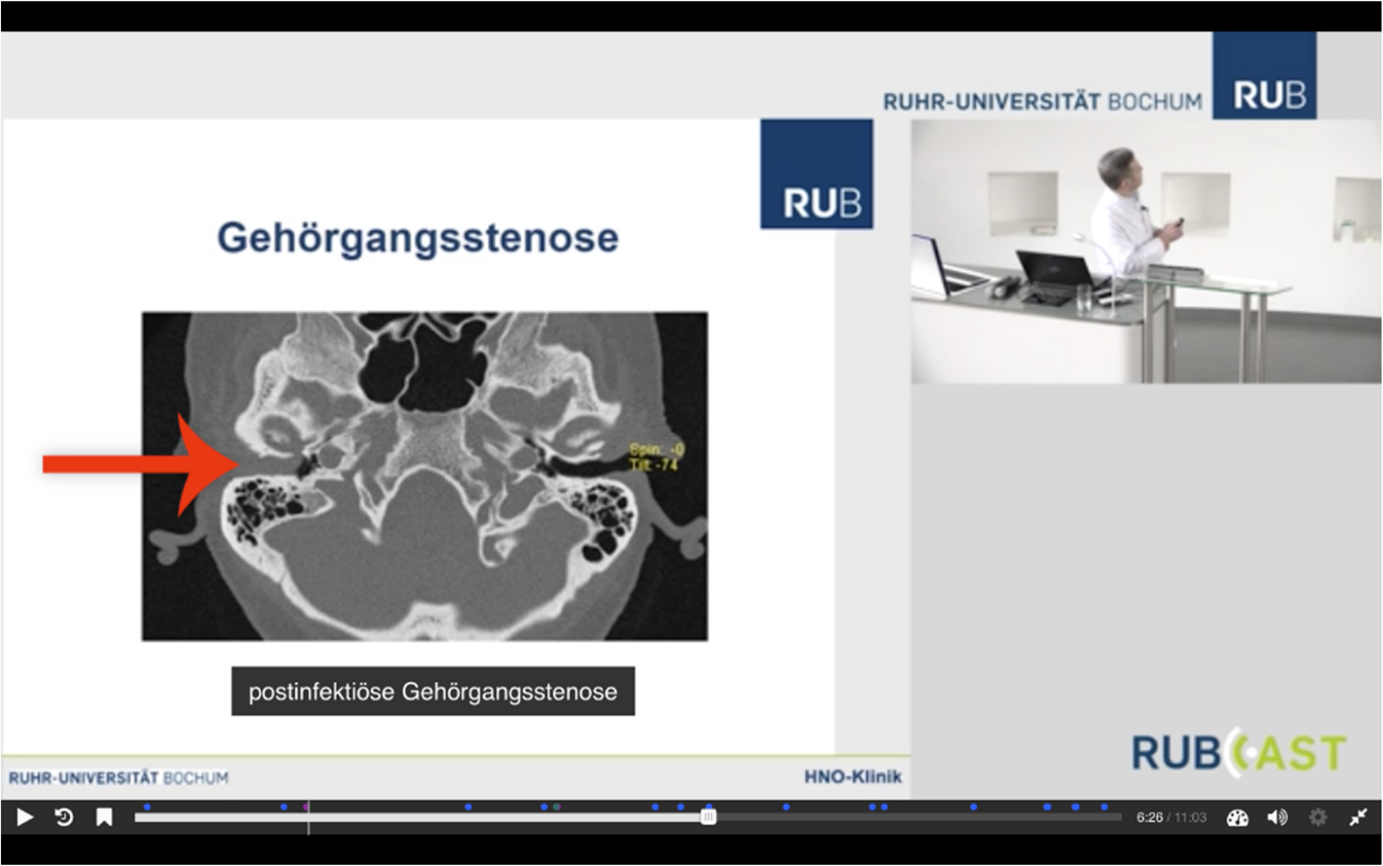
Example for the echo360-compatible design and H5P insert The lecturer is displayed in the upper right corner. To improve quality, slides are not filmed but derived from the presentation file. While pointing on the slide on the wall, the visitor is unable to see the lecturer’s laser pointer. Thus, an arrow was added with the H5P plugin. The caption of the CT scan was also added afterwards with H5P to improve the structure within the video.

1. First, we discussed our regular lecture schedule and adjusted the topics as necessary. Hence, we asked all lecturers to polish their presentations and pay special attention to privacy and copyright issues. Content and structure of the lecture were already aligned to the german national catalogue of learning objectives in medical education (NKLM) beforehand. Thus, we did not change any major issues before the lecture period.
2. In the second step, we performed video and audio recording of all lectures of the Department of Otolaryngology, Head and Neck Surgery at Ruhr University Bochum. After the recording, the material was revised by our in-house media department to fit to the echo360 platform for lecture recordings. We included two consecutive lecture cycles, in which the first cycle served as a backup in case a lecture in the second cycle appeared improvable or if any technical problems occurred during recording or further processing (e.g. sound, video, recording). Additionally, lectures containing introductions, recapitulations or synopses were excluded.
3. To create dependable e-learning content, the revised videos needed to be analysed and rearranged related to a certain topic and unnecessary content needed to be identified. The videos were submitted to two further parallel processing paths to evaluate the minimum time required for any medical doctor participating in this process (Fig. 1). This part of the study was prospectively designed and conducted to reduce bias.
  For path A, five medical professionals watched and analysed all lecture videos at full length, determined the relevant episodes for e-learning, defined the cutting points and the subsequent re-arrangement. Our in-house media department then performed video editing and cut the videos into sections according to the doctor’s instructions.
  In path B, a technician without medical knowledge processed the lecture videos primarily into chunks related to technical aspects (change of slides, headlines, breaks) and afterwards, the video parts were reviewed and sorted by a medical professional with regard to their relevance for e-learning. In this step, the medical professional’s task was practically almost reduced to a binary decision: keeping or discarding the content. Then, the technician rearranged and revised the videos according to the medical professional’s instructions. Videos that did not depict the core topics of the Otolaryngology curriculum, overlapping videos and videos showing discussions in the auditorium or using out-dated guidelines and classifications were excluded in both approaches. An additional reason for exclusion from further processing was patient presentations during the lecture due to personal rights. Generally, we aimed to not exceed a video duration of 15 min [18, 19]. The required working time was documented for each step. Both processing paths were compared and analysed with regards to qualitative and quantitative outcome, options for saving time and necessary working time for medical professionals. In this step, the whole material has to be explored, which was in our case a length of 35 h and 41mins. Therefore, we identified the most labour-intensive step here, offering the opportunity to significantly save working time for medical professionals. The time necessary for this step in each path was analysed separately as well as the number of slides included in the videos as a surrogate parameter for the content.
4. After revising the video material, all topic-related videos were processed with the H5P plugin of our university’s Moodle LMS by a medical professional. Initially, the HTML5 Plugin H5P was developed to create interactive HTML5 contents for websites. The plugin is free and open for use within the MIT license, and it is mobile friendly and supports the content management systems Wordpress, Drupal and the LMS Moodle. Within the various possible content types, we identified the use of interactive videos as the most reasonable for our purposes. In this processing step, we added information to the videos, e.g., links or annotations, when we thought the speaker’s message was unclear or needed to be more precise (Table 1, Fig. 2). We then enriched the content, e.g., with interactive multiple-choice questions, summary slides and opportunities to access further information and deeper insights. The time requirement for this process was also documented. Here, we did not separate the processing into the two previously mentioned paths, as we did not expect any relevant difference between the two paths for this procedure. Only the videos derived from path B were integrated into further processing.

**Table 1:** Drawbacks while processing lecture recordings into chunks which may lead to insufficient quality for teaching and their solutions based on the H5P plugin

Drawback in processing lecture recordings	H5P-based solution
**No laser pointer visible**	insert arrow
**Missing information**	add interactive links, further information
**Mistake in the lecture slide**	insert box with or without text to replace or cover the mistake
**Missing structure**	add summary slides
**Copyright issues of the used images**	replace image
**No educational objectives defined**	add slides defining educational objectives
**Missing assessment of educational objectives**	add interactive quiz

Finally, we again analysed the quantitative and qualitative outcomes. For this task, the number of displayed topics was quantified by comparison with the OTOSource otolaryngology curriculum of the American Academy of Otolaryngology-Head and Neck Surgery [20]. This curriculum is a guideline for specialist training for doctors; thus, not all topics are relevant for medical students. In this context, two categories were excluded from the analysis (“Clinical Fundamentals” and “Business of Medicine”), as these were not included in the educational objectives of our curriculum. In addition, the AAO-HNS curriculum contains surgical training videos, which were also not integrated into further analysis. The total working time for the transformation of the lecture into interactive videos was documented.

The statistical analysis was performed with R Software for statistical computing (The R Foundation). *P*-values were considered significant below the 5% threshold. All analysed data were tested for normality with the Shapiro-Wilk test. Depending on the dataset and question, we used Student’s t-test or the Mann-Whitney U test to test for significance.

## Results

The study included 25 recordings of lectures taken from two consecutive lecture cycles in otolaryngology at Ruhr University Bochum, with a total time of 35 h and 41 min. After the lectures were submitted to our abovementioned exclusion criteria, 10 of the recorded lectures containing 872 min (14 h and 32 min) of video material were included for further editing via Path A and Path B. Beforehand, all recordings were pre-processed by our in-house media department to an echo360 compatible design (step two) showing both the instructor and the slides (Fig. 2).

Accordingly, the pre-processed videos were comparably transferred to processing paths A and B (step three). While in Path A the screening and sorting of video material was performed by a physician, cutting into video chunks was performed by a technician. The physician carefully had to watch the whole material, to set cuts, to generate groups of combinable chunks and finally to instruct the technician. In contrast, in Path B the screening of the video material, and therefore also the important step of decision-making of how the raw video material should be divided into smaller sections, was performed by a technician. In this case, the physician’s task was only discarding the inapplicable videos and optionally generating groups of combinable chunks. Like that, the physician’s task was reduced to almost the binary decision “keep or discard”. Participating physicians also reported that they were able to take this decision partially already in the beginning of some chunks and could resign to watch the remaining video. On average, the videos generated via Path B were 32 s shorter than videos generated via Path A.

Overall, step three required a total time of 697 min (11 h and 37 min) for Path A vs. 993 min (16 h 33 min) for Path B and 70 min (95%-CI: 58–82 min) per lecture for Path A vs. 99 min (95%-CI: 80–119 min) per lecture for Path B. For technicians, the summarized working time in Path A was 186 min and 620 min in Path B. In contrast, the summarized working time of medical professionals was 511 min in Path A and 373 min in Path B. The average working time for medical professionals was 37 min (95%-CI: 22–52 min) per lecture for Path B vs. 51 min (95%-CI: 41–62 min) per lecture for Path A.

Comparing the video processing of both paths for step three in general, Path A is less time consuming than Path B. But even though Path B required a 433 min (7 h 14 min) longer processing time for the media department (43 min/lecture, 95% CI: 35–52 min, *p* < 0.001), the outsourcing of sorting the video material in Path B also provides the opportunity of saving time for medical professionals, who saved 138 min (2 h and 18 min) of processing time in total (14 min/lecture, 95%-CI: − 7-35 min, *p* = 0.028) (Table 2).

**Table 2:** Overview of working time and length of resulting videos for Path A and B separately and their difference. Time in minutes

Working time for video processing [min]	Path A	Path B	Path B - Path A
Working time technician	186.35	620	433.65
Working time medical professional without H5P	511	373.2	− 137.8
Cummulated workingtime without H5P	697.28	993	295.72
Working time medical professional for revision with H5P	550.48	550.48	0
Overall working time	1247.83	1543.68	295.85
**Resulting video material**			
Accumulated video length after video editing [min]	399.73	359.75	− 39.98
Mean videolength [min]	5.88	4.73	−1.15
Number of resulting videos	76	68	−8
Number of lecture slides	375	318	− 57

**Fig. 3:**
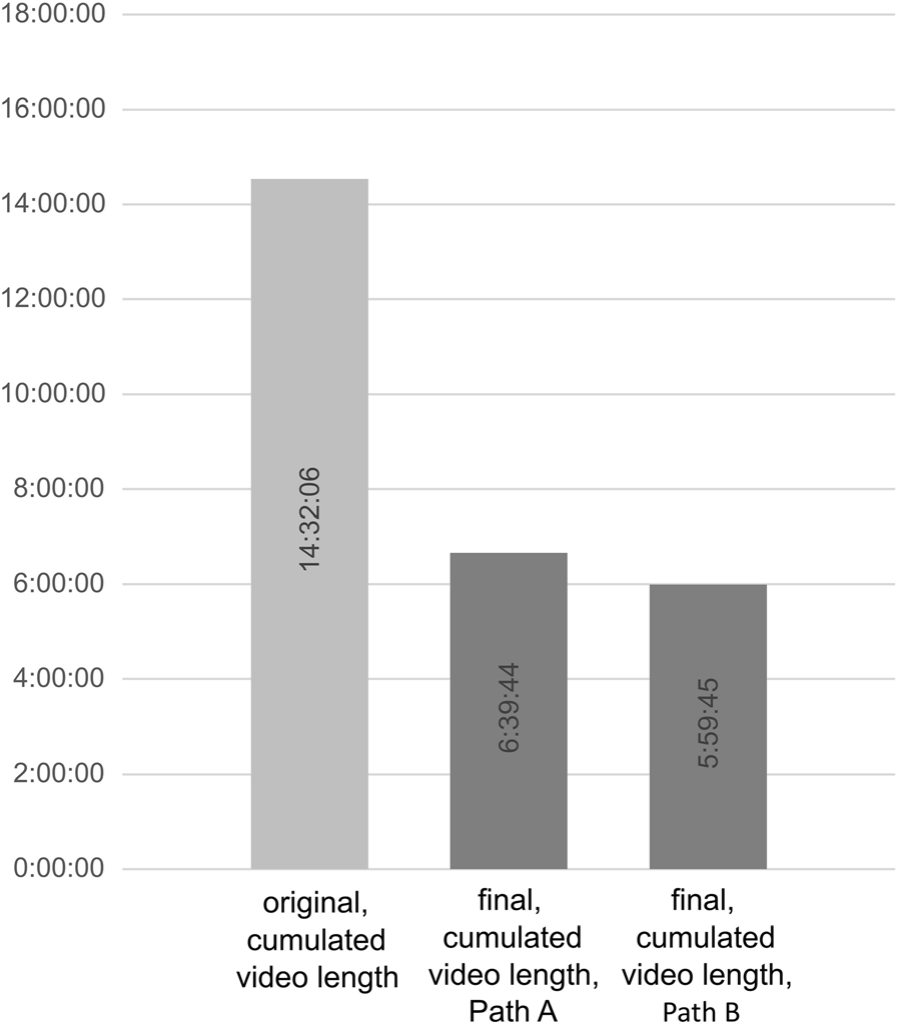
Summary of video lengths and number of resulting videos from the recorded lectures (*n* = 10) before and after processing via Path A vs. Path B

Afterwards, for Path A, the accumulated videos totalled 399.73 min (6 h 39 min and 44 s) and were subdivided into 76 videos, and for Path B, the accumulated videos totalled 359.75 min (5 h 59 min and 45 s) and were subdivided into 68 videos (Fig. 3). Among these videos, video lengths ranged from 35 s to 31 min and 32 s. The mean video length was 5 min and 53 s for Path A and 4 min and 44 s for Path B. Thus, the difference in video length between Path A and Path B was 69 s per video and 3 min and 59 s per lecture. Alongside the analysis by length, video content generated via Path A and Path B was analysed by comparing the number of lecture slides contained in both paths serving as a surrogate. Overall, Path A contained 375 lecture slides, while Path B contained only 318 lecture slides. The mean difference per lecture was 5,7 lecture slides (Table 2).

After video processing, the final videos were analysed by a medical professional again with respect to their completeness regarding each topic and revised with H5P tools (step four). Regarding the highly overlapping content, the difference between both groups in this step to be neither relevant nor meaningful for the analysis. Thus, we performed this step only once and considered the additional working time to be the same for both paths. When useful content was missing, the relevant information was supplemented by links or additional lecture slides. Furthermore, we included marks on pictures to which the lecturer pointed during the lecture, so the laser pointer became visible in the video as well. To increase the learning effect, we then enriched the content with interactive multiple-choice questions and quizzes (see Table 1). This process required 550 min (9 h 10 min) of working time in total, with a mean time of 55 min per lecture (95% CI: 24–86 min) or 7 min 15 s per video by a medical professional (Table 2).

**Fig. 4:**
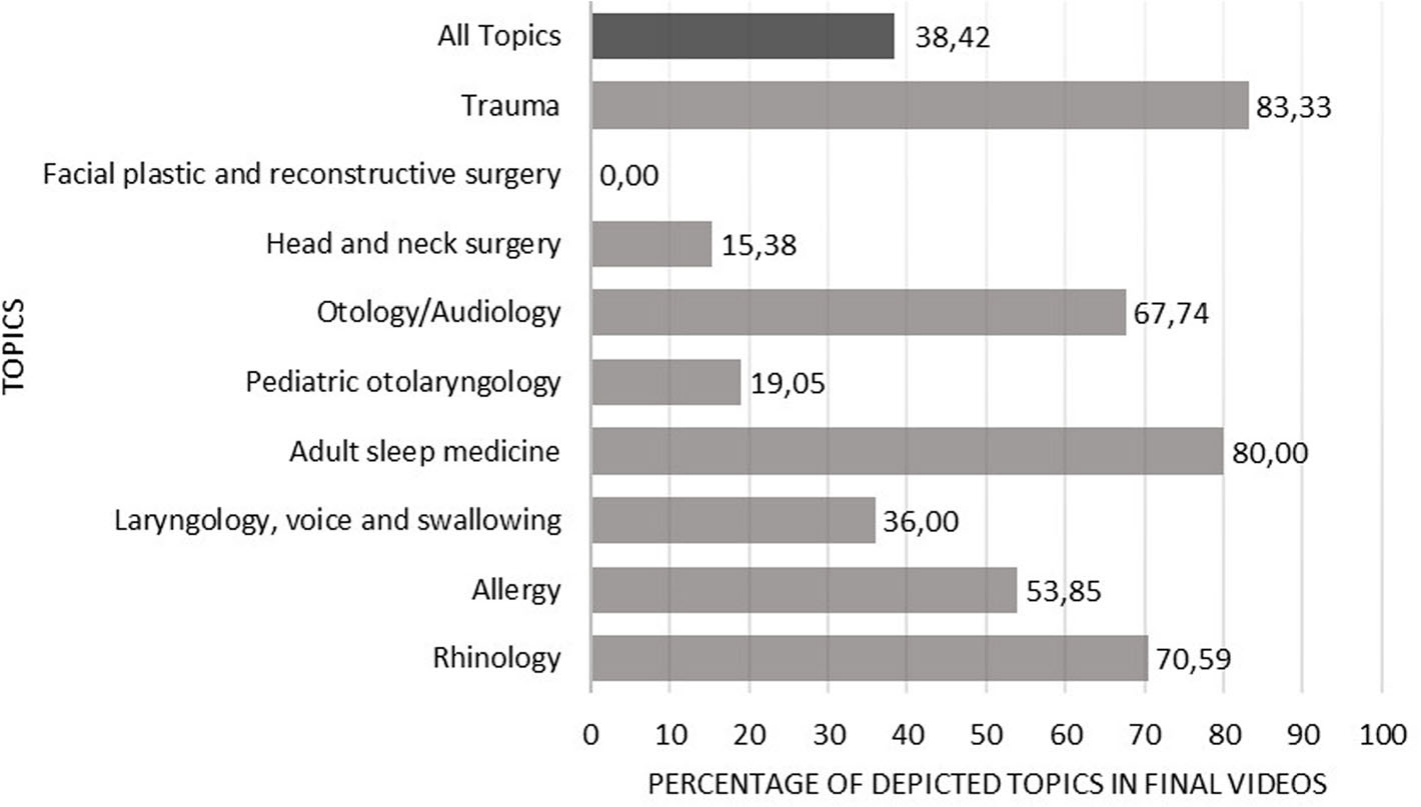
Percentage of depicted topics in comparison with OTOSource otolaryngology curriculum of the American Academy of Otolaryngology-Head and Neck Surgery

The content of the resulting videos was then compared with the OTOSource otolaryngology curriculum of the AAO-HNS to assess the number of depicted topics. In total, the 9 remaining categories contained 190 different topics, of which we depicted 73 topics in interactive videos online (38,42%). There were major differences between categories; “trauma” was the best represented category with 83,33% coverage, while “facial plastic and reconstructive surgery” was not represented at all. The median number of depicted topics per category was 47,3% (Fig. 4).

**Fig. 5:**
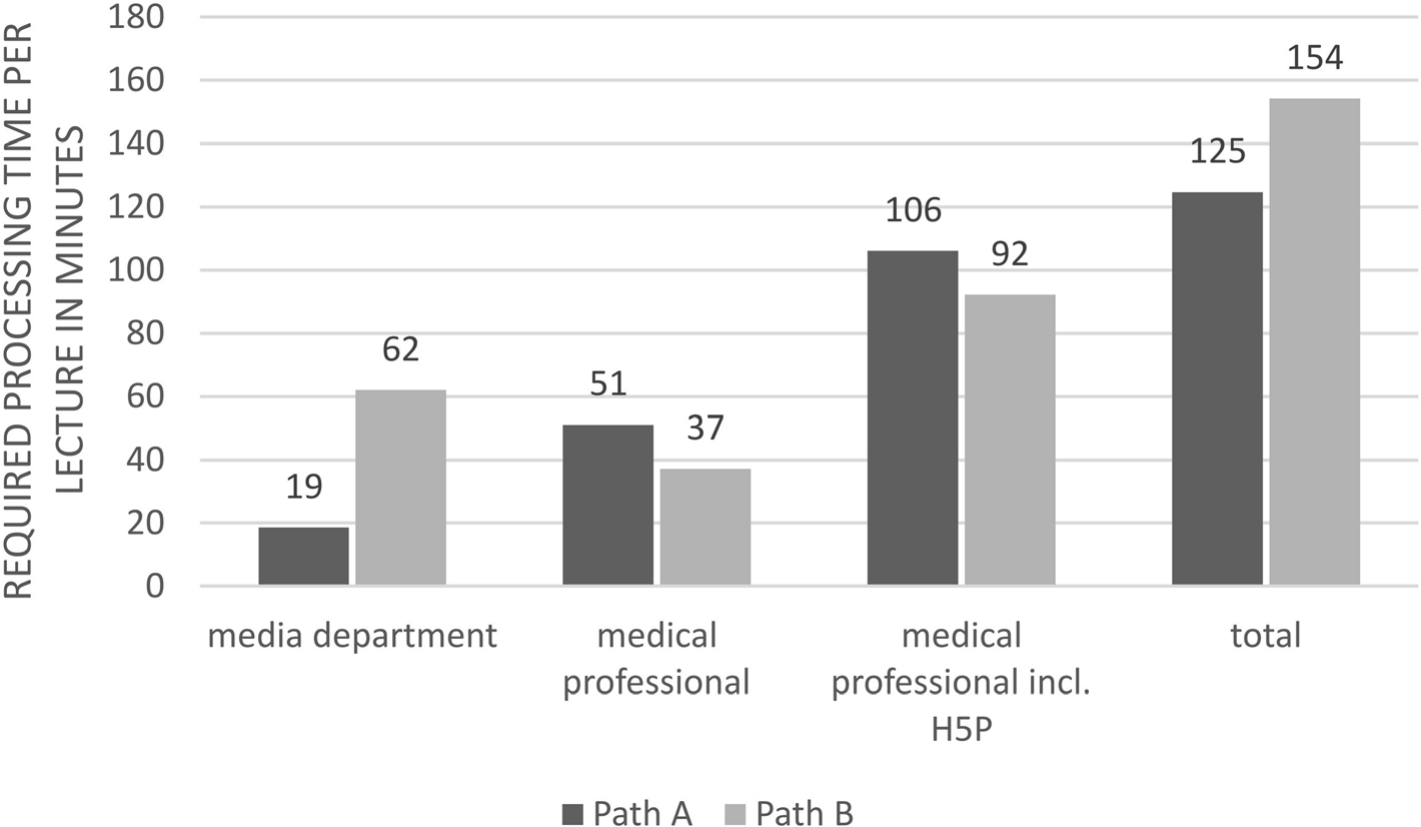
Required processing time per lecture for the different processing steps and in total (Path A vs. Path B)

Overall, the processing of 14 h and 32 min (872 min =10 lectures) of raw video material required 20 h 48 min (1248 min) for Path A vs. 25 h and 44 min (1544 min) for Path B and resulted in 6 h and 16 min (376 min) of teaching videos. The average total working time per lecture was 125 min (95% CI: 92–157 min) for Path A vs. 154 min (95% CI: 107–201 min) for Path B, which resulted in a medium time savings of 30 min with the use of Path B (*p* > 0.05) (Fig. 5).

## Discussion

Our work presents a comprehensive, fast-tracked approach for transforming a pre-existing, lecture-based otolaryngology curriculum into structured digitized content within a Moodle learning management system. In our case, this content was intended to be integrated into a flexible flipped classroom-supported framework. As we combined currently available technical processing and open-source software, our content development strategy could be easily transferred to other lecture-based curricula and is not limited to otolaryngology. By comparing two different paths of video processing, we tracked two ways for digitizing whole lecture cycles which as well reduce the required working time for medical professionals.

### Video processing

While creating suitable multimedia content is a key challenge and is usually time-consuming, especially for flipped classroom frameworks, we demonstrated that, following our approach, a total working time of 20 h 48 min (1248 min) for Path A or 25 h and 44 min (1544 min) for Path B was sufficient to process a complete curriculum [18, 21].

Considering the daily duties of medical professionals in clinical practice, the study focused particular attention on achieving a minimal time requirement for medical professionals by comparing two parallel workflows, which showed that it is possible to save considerable time for medical professionals by outsourcing major parts of video editing.

Because the pivotal, most time-consuming step of video processing appeared to be the editing of the raw video material into topic-related sections (screening, sorting, cutting), we observed the highest potential for a time-saving strategy for this task.

Our work showed that, despite interpersonal differences in technical skills, this step required less working time when the screening of raw material was completed by a medical professional (Path A), which might be explained by the fact that medical professionals were able to differentiate and organize content. Due to their expertise, they knew which topics were less relevant, while staff members of the media department were confronted with otolaryngology-specific topics for the first time and therefore preferred a more technical approach that involved looking at speech pauses or slide organization. Hence, the overall required working time was also shorter (by 296 min) when the video chunks were subdivided by a medical professional (20 h 48 min (1248 min) in Path A vs. 25 h and 44 min (1544 min) in Path B). Regardless, we observed a significant time savings of 138 min (2 h and 18 min) for medical professionals when the sectioning of the videos was performed by the media department (Path B).

We believe that the pre-processing in Path B accelerated decision-making by the medical professional, as the technician had already deconstructed the lecture’s superstructure and provided “decision support” that the medical professional could accept or decline. Even though the overall required working time is longer in this approach (Path B), the extent of the time savings for physicians offers advantages. These advantages are particularly evident since otolaryngology is one of the smaller clinical disciplines in pregraduate medical education, which leads to their being relatively limited overall working time for both paths. Considering processing in larger clinical disciplines with our approach, such as internal medicine or general surgery, the whole process should require much more work time. Thus, outsourcing the sectioning to the media department (Path B) may vastly increase the observed time savings for medical professionals.

### Video content and quality

Because one could assume that by doing so the level of content quality decreases, we precisely analysed the resulting videos of both paths with regards to differences in quality and quantity.

On average, videos generated by Path B were shorter than those subsectioned by medical professionals, and they contained approximately the same content (Path A). This is because the videos in Path B were partly composed of more short video fragments, while the videos in Path A usually consisted of one continuous video; thus, they contained more linking words and phrases, as well as context-specific details. In this regard, not only the video length but also the number of depicted lecture slides were higher in Path A, which contained more lecture slides with, for example, deeper insights into the classification of diseases and therapies, which are not necessarily relevant for students to pass the final exam but complement clinically relevant aspects. Whenever reasonable, this supplemental material can also be included in videos generated via Path B in form of links, pictures and other references. Consequently, there was no perceptible loss of video quality or content in both parts.

### Curriculum design / flipped classrooms

As indicated by Wagner et al. [18], students seem to prefer a 30% flipped lecture component; thus, not all videos should be used for teaching during one lecture cycle.

In this study, we nevertheless transformed all topics into flipped classroom compatible videos to enable a variety of possible flipped classroom lectures. Furthermore, by making all videos accessible online, students additionally have the opportunity to independently make up missed lectures or to repeat single topics. If these effects are not intended, there could be additional time savings by only converting single topics into flipped-classroom content. In our case, we preferred the flexible approach for a better understanding of appropriate topics for flipped classrooms in otolaryngology. For the practical implementation, we therefore highlighted and cross-linked the videos related to a certain flipped classroom event.

Because this study does not describe the creation of a curriculum from the beginning, the preparation time for creating the classical face-to-face lecture itself was not integrated into the analysis and would also need to be considered if the face-to-face lecture has not already been created. Lecture recordings as a simple reproduction of a lecture are neither innovative nor ground-breaking and their benefit for the learner remains under a controversial discussion. Whereas some authors state that real lectures are better in experience and learning outcome [22, 23], others report high demand and intensive utilization of lecture recordings [24, 25]. A current moderate position concerning this issue is to see the recordings as a supplement to the lecture that may be useful e.g. in cases of sickness, language barriers or scheduling conflicts [26]. Here, we see the main benefit of using H5P tools as we could add, e.g., annotations or missing information, to the pre-existing lecture recordings whenever such material appeared to be useful. Using chunks of the whole lecture recordings only, we were also able to move information within the framework and concentrate the content (e.g. with H5P-generated summary slides). Thus, we think that the educational value of the lecture recording could be significantly improved at this step. Modifying the lecture in advance, before recording, would of course be the superior approach but would again result in increased working time.

The effectiveness of flipped classrooms learning designs was not a subject of this work since we investigated the very first step of creating a flipped classroom curriculum. Recent meta-analysis, trying to summarize the widespread literature about flipped classrooms, agree that current evidence suggests an improvement compared to classical teaching strategies while strong evidence and the analysis of long term effects are still lacking [9, 11].

### Cost-effectiveness

While many authors investigated the educational value for flipped classroom designs, its cost-effectiveness is usually beyond the scope of most publications [27]. As reported by McLaughlin and colleagues, their faculty registrated an increase of 127% more time for preparing their flipped classroom compared to a traditional classroom [14]. In our case, this comparison was unfortunately not meaningful. Anyway, we agree with other authors, that flipped classrooms require resources and expertise, at least in the beginning [17, 27]. To our best knowledge, here we present a detailed analysis of working time for the participating professions in the beginning of a flipped classroom curriculum for the first time in literature. We think that our proposed approach is a suitable, time-saving strategy to transform a whole curriculum into a video-based flipped classroom framework. Within this, the outsourcing of major parts of video processing leads to relevant time savings for medical professionals while providing equal content. The use of H5P tools to improve content was, in our hands, a both meaningful and effective strategy to counter the weaknesses of deconstructing lecture recordings and reduce final video editing to a minimum.

## Conclusions

Transforming lecture recordings into online teaching and learning content can be a fast-track strategy to create a solid groundwork for blended learning approaches. The use of H5P interactive tools via Moodle LMS provides a major procedural benefit by allowing the easy adjustment of pre-existing video material into suitable online content. Reasonably outsourcing work to technicians based on our strategy can significantly reduce the working time of medical professionals without decreasing the quality of learning content. The workflow presented here can be used as a flexible approach for flipped classroom frameworks or other blended learning strategies where interactive videos are applicable.

## Data Availability

The datasets generated and/or analysed during the current study are not publicly available due to privacy guidelines but are available from the corresponding author on reasonable request.

## Abbreviations

AAO-HNS: American Academy of Otolaryngology, Head and Neck Surgery
CI: Confidence Interval
FC: Flipped Classroom
LMS: Learning Management System

## References

[1] Prober CG, Khan S (2013). Medical education reimagined: a call to action. Acad Med.

[2] Ishman SL, Stewart CM, Senser E, Stewart RW, Stanley J, Stierer KD (2015). Qualitative synthesis and systematic review of otolaryngology in undergraduate medical education. Laryngoscope.

[3] Lin Y, Zhu Y, Chen C, Wang W, Chen T, Li T (2017). Facing the challenges in ophthalmology clerkship teaching: is flipped classroom the answer?. PLoS One.

[4] Cheng X, Ka Ho Lee K, Chang EY, Yang X (2016). The “flipped classroom” approach: stimulating positive learning attitudes and improving mastery of histology among medical students: flipped classroom in histology course in China. Anat Sci Educ.

[5] Dombrowski T, Wrobel C, Dazert S, Volkenstein S (2018). Flipped classroom frameworks improve efficacy in undergraduate practical courses - a quasi-randomized pilot study in otorhinolaryngology. BMC Med Educ..

[6] Ferrer-Torregrosa J, Jiménez-Rodríguez MÁ, Torralba-Estelles J, Garzón-Farinós F, Pérez-Bermejo M, Fernández-Ehrling N (2016). Distance learning ects and flipped classroom in the anatomy learning: comparative study of the use of augmented reality, video and notes. BMC Med Educ..

[7] Lew EK (2016). Creating a contemporary clerkship curriculum: the flipped classroom model in emergency medicine. Int J Emerg Med.

[8] Sánchez JC, López-Zapata DF, Pinzón ÓA, García AM, Morales MD, Trujillo SE (2020). Effect of flipped classroom methodology on the student performance of gastrointestinal and renal physiology entrants and repeaters. BMC Med Educ..

[9] Chen F, Lui AM, Martinelli SM (2017). A systematic review of the effectiveness of flipped classrooms in medical education. Med Educ.

[10] Prober CG, Heath C (2012). Lecture halls without lectures — a proposal for medical education. N Engl J Med.

[11] Hew KF, Lo CK. Flipped classroom improves student learning in health professions education: a meta-analysis. BMC Med Educ. 2018;18. 10.1186/s12909-018-1144-z. 10.1186/s12909-018-1144-z PMC5855972 29544495

[12] Lockman K, Haines ST, McPherson ML (2017). Improved learning outcomes after flipping a therapeutics module: results of a controlled trial. Acad Med.

[13] Chen K-S, Monrouxe L, Lu Y-H, Jenq C-C, Chang Y-J, Chang Y-C (2018). Academic outcomes of flipped classroom learning: a meta-analysis. Med Educ.

[14] McLaughlin JE, Roth MT, Glatt DM, Gharkholonarehe N, Davidson CA, Griffin LM (2014). The flipped classroom: a course redesign to Foster learning and engagement in a health professions school. Acad Med.

[15] Blair RA, Caton JB, Hamnvik OR (2020). A flipped classroom in graduate medical education. Clin Teach.

[16] Tolks D, Schäfer C, Raupach T, Kruse L, Sarikas A, Gerhardt-Szép S (2016). An introduction to the inverted flipped classroom model in education and advanced training in medicine and in the healthcare professions. GMS J Med Educ.

[17] Vanneman M, Baker K, Saddawi-Konefka D (2017). Studies on the effectiveness of flipped classrooms: are we comparing apples to apples?. Med Educ.

[18] Wagner D, Laforge P, Cripps D. Lecture material retention: a first trial report on flipped classroom strategies in electronic systems engineering at the University of Regina. Montreal: CEEA13; 2013.

[19] Sharma N, Lau CS, Doherty I, Harbutt D (2015). How we flipped the medical classroom. Med Teach.

[20] AAO-HNS. Otosource - comprehensive otolaryngology curriculum. 2018. https://www.otosource.org. Accessed 31 May 2021.

[21] Moffett J (2015). Twelve tips for “flipping” the classroom. Med Teach.

[22] Cardall S, Krupat E, Ulrich M (2008). Live lecture versus video-recorded lecture: are students voting with their feet?. Acad Med.

[23] Bacro TRH, Gebregziabher M, Fitzharris TP. Evaluation of a lecture recording system in a medical curriculum. Anat Sci Educ. 2010;3(6):300-8. 10.1002/ase.183 20954266

[24] Johnston ANB, Massa H, Burne THJ (2013). Digital lecture recording: a cautionary tale. Nurse Educ Pract.

[25] Gupta A, Saks NS (2013). Exploring medical student decisions regarding attending live lectures and using recorded lectures. Med Teach.

[26] Kwiatkowski AC, Demirbilek M (2016). Investigating veterinary medicine faculty perceptions of lecture capture: issues, concerns, and promises. J Vet Med Educ.

[27] Chen F, Lui AM, Martinelli SM (2018). In response to Vanneman et al. on ‘Studies on the effectiveness of flipped classrooms.’. Med Educ.

